# Special Section Guest Editorial: Advances in Terahertz Biomedical Science and Applications

**DOI:** 10.1117/1.JBO.26.4.043001

**Published:** 2021-04-28

**Authors:** Kirill I. Zaytsev, Vladimir N. Kurlov, Maksim Skorobogatiy, Igor V. Reshetov, Valery V. Tuchin

**Affiliations:** a Prokhorov General Physics Institute of the Russian Academy of Sciences, Russia; b Sechenov University, Institute for Regenerative Medicine, Russia; c Bauman Moscow State Technical University, Russia; d Institute of Solid State Physics of the Russian Academy of Sciences, Russia; e Polytechnique Montreal, Department of Engineering Physics, Canada; f Sechenov University, Institute for Cluster Oncology, Russia; g Academy of Postgraduate Education FSCC, FMBA, Russia; h Saratov State University, Russia; i Institute of Precision Mechanics and Control of the Russian Academy of Sciences, Russia; j National Research Tomsk State University, Russia

## Abstract

The editorial introduces a special section of the Journal of Biomedical Optics on Advances in Terahertz Biomedical Science and Applications. This special section includes one review and ten research papers addressing the complex challenges of terahertz biophotonics and related areas of biomedical optics.

With rapid progress in terahertz (THz) technology, quite portable and ergonomic THz spectroscopy and imaging systems emerge, thus, pushing research and engineering efforts into applying THz technology in various demanded branches of applied physics and optics, including biomedical optics.^1^^–^^6^ The strong sensitivity of THz waves to the content and state (free or bound) of tissue water^7^ leads to novel opportunities in such applications as label-free diagnosis of malignant and benign neoplasms with different nosology and localization,^8^^,^^9^ diabetes mellitus,^10^^–^^12^ tissue viability^13^ and traumatic injuries,^14^ and even therapy for cancers and inflammatory diseases.^15^^,^^16^ Inspired by such rapid progress, the *Journal of Biomedical Optics* (JBO) has published this special section of papers to capture the most recent advances in THz technology and innovative THz instruments and methods in biology and medicine. Moreover, a few of the papers in this special section are dedicated to similar biomedical applications of novel optical tools from the neighboring infrared (IR) range.

In the review paper by A.I. Nikitkina et al., entitled “Terahertz radiation and the skin: a review” (URL: https://doi.org/10.1117/1.JBO.26.4.043005), modern research results in the area of THz-wave–skin interactions are summarized, considering both diagnostic and therapeutic applications. Indeed, due to quite a limited depth of THz-wave penetration in tissues along with the simplicity of THz measurements of the skin, it remains the most studied localization in the THz range, with a numerous emerging clinical applications.

Two papers of the special section consider modern problems of oncodiagnosis. In the research article “Development of oral cancer tissue-mimicking phantom based on polyvinyl chloride plastisol and graphite for terahertz frequencies” (URL: https://doi.org/10.1117/1.JBO.25.12.123002), T. Zhang et al. introduced a new type of a water-free tissue-mimicking phantom for THz biophotonics. This phantom is based on graphite powders embedded into a polyvinyl chloride plastisol matrix. The effective THz optical properties of such a phantom (including refractive index, absorption, and dispersion) can be managed in a wide range by changing its composition, thus allowing to mimic the THz optical properties of various biological tissues. Obviously, development of such novel low-cost and reproducible tissue-mimicking phantoms is an important problem of THz biophotonics. Second, capabilities of mid-IR interband cascade laser in surgical thermal ablation of normal fibroblasts and primary undifferentiated pleomorphic sarcoma tumor cells were demonstrated by E. Larson and co-authors in the paper “Mid-infrared absorption by soft tissue sarcoma and cell ablation utilizing a mid-infrared interband cascade laser” (URL: https://doi.org/10.1117/1.JBO.26.4.043012).

Two research works consider applications of THz technology in studying glycated tissues and blood, aimed at diabetes mellitus diagnosis. In one paper by A.A. Lykina et al., entitled “Terahertz spectroscopy of diabetic and non-diabetic human blood plasma pellets” (URL: https://doi.org/10.1117/1.JBO.26.4.043006), an ability to differentiate between lyophilized (dehydrated) pellets of diabetic and non-diabetic human blood plasma using the THz pulsed spectroscopy was demonstrated. In another paper by A.A. Lykina et al., entitled “Terahertz high resolution spectroscopy of thermal decomposition gas products of blood plasma and kidney tissue pellets” (URL: https://doi.org/10.1117/1.JBO.26.4.043008), content of the thermal-decomposition gas products of diabetic and non-diabetic dried blood plasma and kidney tissues was studied, involving methods of high-resolution gas spectroscopy at THz frequencies. The authors show that such gas spectroscopy in the THz range also yields useful information for the differentiation between diabetic and non-diabetic samples, where acetone serves as a main marker of diabetic blood and tissues.

Completely novel applications of THz pulsed spectroscopy (or of, so-called, THz time-of-flight tomography^17^^–^^19^) were discovered by A.K. Zotov et al. in their article “*In situ* terahertz monitoring of an ice ball formation during tissue cryosurgery: a feasibility test” (URL: https://doi.org/10.1117/1.JBO.26.4.043003). Using quite different THz dielectric responses of intact tissues (either *in vivo* or freshly excised *ex vivo*) and frozen ones,^20^ they highlighted an ability for intraoperative measurements of ice ball formation during tissue cryosurgery. Namely, THz pulsed spectroscopy was found to be able to detect the interface between intact and frozen tissues at a depth of hundreds of microns. Thereby, THz technology has potential for estimating the freezing depth of tissue in different areas of modern cryosurgery.

Two papers of the special section are dedicated to the THz sensing of corneal properties. In a research article by E.N. Iomdina and co-authors, entitled “Terahertz scanning of the rabbit cornea with experimental UVB-induced damage: *in vivo* assessment of hydration and its verification” (URL: https://doi.org/10.1117/1.JBO.26.4.043010), the high sensitivity of the THz-wavelength to the content of water in tissues was exploited for studying pathological changes in the cornea caused by ultraviolet light exposure. The observed results of the rabbit cornea measurements in the THz range were verified by optical coherence tomography. They highlighted an ability for *in vivo* contactless estimation of the cornea hydration (and, thus, related pathologies) based on its THz reflectivity. In turn, in a paper by L. Ke et al., entitled “Terahertz spectroscopy analysis of human corneal sublayers” (URL: https://doi.org/10.1117/1.JBO.26.4.043011), a method for sensing human corneal composition at different depths (with a focus on the epithelium and the stromal layer) was proposed, relying on high-sensitivity THz spectroscopy. Using novel approaches to collecting and processing data, the authors studied THz responses of human corneas at different edema stages. The observed results demonstrated that the epithelium layer of cornea acts as a barrier that maintains the hydration level of the stroma; while at the final stages of edema, the epithelium lost its barrier properties. The epithelium quality can be used to predict the level of corneal swelling.

New applications of spectroscopic and imaging systems from the IR range, which is neighboring to THz, are also considered in this special section. C. Healy and co-authors, in their paper “Globally deployed COVID-19 fever screening devices using infrared thermographs consistently normalize high readings to afebrile range” (URL: https://doi.org/10.1117/1.JBO.26.4.043009), addressed a problem of human temperature measurement using IR thermal imaging systems, with an emphasis on diagnosis of possible infectious disease transmission, including COVID-19. A systematic study of the IR spectra of insulin involving model insulin specimens was carried out by S. Delbeck and H.M. Heise, in their article “Systematic stability testing of insulins as representative biopharmaceuticals by using ATR FTIR-spectroscopy with focus on quality assurance” (URL: https://doi.org/10.1117/1.JBO.26.4.043007). Insulin specimens were stored at different temperatures (i.e., 0°C, 20°C, and 37°C) for up to three months, while their weekly IR spectroscopic measurements yielded monitoring of the hormonal secondary structural changes, which correlated negatively with the insulin bioactivity. The observed results revealed that IR spectroscopy holds potential for rapid and reliable analysis of the secondary structural changes within insulin molecules and, thus, is capable of insulin quality control. Finally, Y. Zhu and co-authors, in their article “Dual short wavelength infrared transillumination/reflectance mode imaging for caries detection” (URL: https://doi.org/10.1117/1.JBO.26.4.043004), developed a system for simultaneous acquisition of both the short-wavelength infrared reflectance and occlusal transillumination images of lesions on tooth proximal and occlusal surfaces, which is aimed at differentiating between shallow and deep occlusal lesions.

The content of the special section includes selected papers presented at the Annual International Conference Saratov Fall Meeting 2020 (SFM’20),^21^ which was held in Saratov State University (Saratov, Russia) in a mixed (online and onsite) format. All guest editors of this JBO special section were involved in this meeting as members of the organizing and program committees, with Prof. V.V. Tuchin chairing SFM’20. Actively supported by SPIE, this conference brings together researchers from different countries and provides a platform for scientific collaboration, as well as an opportunity for students and young researchers to meet world-class scientists.
